# A chromatographic approach to development of 5-aminosalicylate/folic acid fixed-dose combinations for treatment of Crohn’s disease and ulcerative colitis

**DOI:** 10.1038/s41598-020-77654-2

**Published:** 2020-11-30

**Authors:** Mario-Livio Jeličić, Edvin Brusač, Daniela Amidžić Klarić, Biljana Nigović, Nikša Turk, Ana Mornar

**Affiliations:** 1 Faculty of Pharmacy and Biochemistry, University of Zagreb, A. Kovačića 1, 10000 Zagreb, Croatia; 2 Clinical Hospital Center Zagreb, Kišpatićeva 12, 10000 Zagreb, Croatia

**Keywords:** Medicinal chemistry, Analytical chemistry

## Abstract

Medication adherence is an important factor in inflammatory bowel disease therapy, which includes regular supplementation of malabsorbed vitamins. Absorption of folic acid is limited due to the damaging of the gastrointestinal tract, which can increase the chances to develop megaloblastic anaemia and colorectal cancer. In this work, 5-aminosalicylates (mesalazine, balsalazide, sulfasalazine and olsalazine) and folic acid were characterized regarding their pharmacokinetic related properties (hydrophobicity, phospholipid and plasma protein binding) using the biomimetic chromatographic approach. Despite the high binding percentage of 5-aminosalicylates for human serum albumin (> 61.44%), results have shown that folic acid binding to human serum albumin protein is far greater (69.40%) compared to α1-acid-glycoprotein (3.45%). Frontal analysis and zonal elution studies were conducted to provide an insight into the binding of folic acid to human serum albumin and potential competition with 5-aminosalicylates. The analytical method for the simultaneous determination of assay in proposed fixed-dose combinations was developed and validated according to ICH Q2 (R1) and FDA method validation guidelines. Separation of all compounds was achieved within 16 min with satisfactory resolution (*R*_s_ > 3.67) using the XBridge Phenyl column (150 × 4.6 mm, 3.5 µm). High linearity (*r* > 0.9997) and precision (RSD < 2.29%) was obtained, whilst all recoveries were within the regulatory defined range by British (100.0 ± 5.0%) and United States Pharmacopeia (100.0 ± 10.0%).

## Introduction

**Figure 1:**
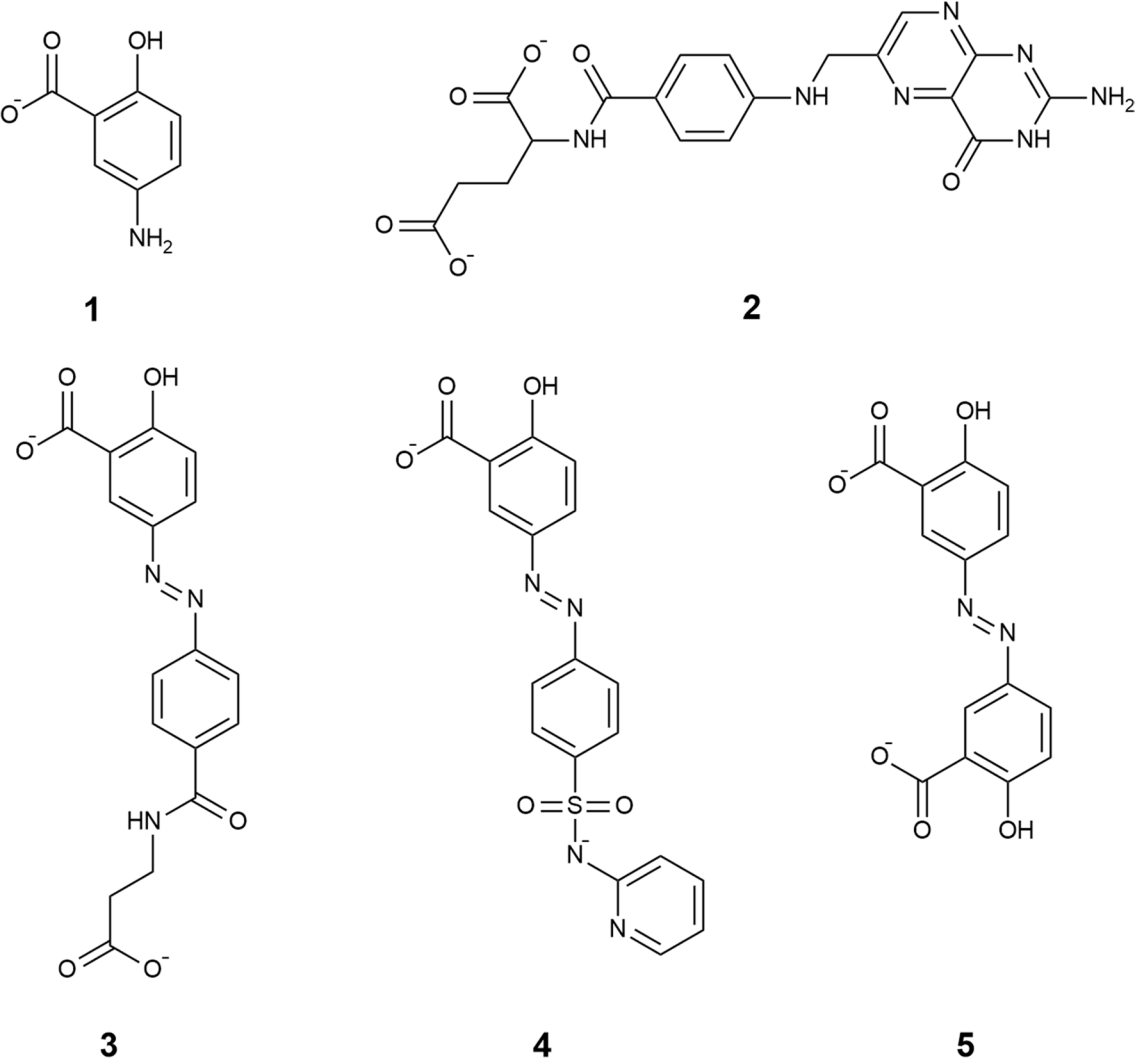
Structures of: MSZ (1), FA (2), BSZ (3), SASP (4) and OSZ (5) at physiological pH (pH = 7.4).

Inflammatory bowel diseases (IBD), including Crohn’s disease and ulcerative colitis, are chronic inflammations of the gastrointestinal tract. The incidence and prevalence of IBD are increasing worldwide, however, the etiology is not precisely defined^1^. Although therapeutic goals to achieve mucosal healing and deep remission require early therapy with novel biologics and small molecules, there are subgroups of patients that can benefit only from 5-aminosalicylate treatment^2^. 5-aminosalicylates are a group of anti-inflammatory drugs including mesalazine (MSZ) and its prodrugs balsalazide (BSZ), sulfasalazine (SASP) and olsalazine (OSZ) (Fig. 1).

Daily intake of multiple drugs during the therapy of IBD is mandatory due to the malabsorption of vitamins, mostly as a consequence of damaged tissue in the gastrointestinal tract. Deficiency of important vitamins, such as folic acid (FA) (Fig. 1), which is necessary for the normal functioning of the human body, can lead to the development of megaloblastic anaemia and colorectal cancer^3,4^. With the rise of fixed-dose combinations (FDCs) the potential risk to fail the adherence should be minimized^5^. FDC presents a single formulation containing two or more different active pharmaceutical ingredients, where the safety and efficacy of the novel combination is not compromised compared to every product separately^6^.

To improve the quality of IBD patients’ lives, FDCs are proposed in collaboration with Clinical Hospital Centre Zagreb. The four formulations would consist of each 5-aminosalicylate combined with FA (MSZ + FA, BSZ + FA, SASP + FA, OSZ + FA). Proposed FDCs would contain therapeutic doses of each drug in order to ensure the maintenance of the remission state and to supplement the malabsorbed FA.

The development of FDCs is driven by several factors, among which is the investigation of the physicochemical properties of each drug. Reversed-phase columns can offer a great alternative to the conventional methods for hydrophobicity determination, where on the other hand, biomimetic columns with immobilized endogenous structures, such as artificial membrane (IAM), human serum albumin (HSA) and α1-acid-glycoprotein (AGP) can give additional information regarding the drug behaviour once it enters the human body, such as cell membrane permeability and plasma protein binding (PPB)^7^. The pharmacokinetics of drugs is mainly influenced by the interaction of drugs with the plasma proteins. HSA, being the most abundant protein in plasma (35–50 g/L), and AGP (0.6–1.2 g/L) are major binding agents for most of the drugs^8,9^. Frontal analysis and zonal elution studies are well-known approaches in characterization of drug binding to plasma proteins which include the application of high-performance affinity chromatography (HPAC) with the use of biomimetic columns to monitor binding behaviour of drugs^10^. So far, it has shown to be a successful method for getting an insight into drug binding and possible competition on both HSA and AGP^8,9^. The ability of high throughput biomimetic measurements reduces the required time and resources in gaining important information about active pharmaceutical ingredients and their interactions early on in the drug development process.

From the analytical point of view, each developed formulation requires sufficient quality control methods. Various techniques such as spectrophotometry^11^, HPLC^12–15^, differential pulse voltammetry^16^ resonance light scattering method^17^ and high-performance thin-layer chromatography^18^ were reported through the years for assay determination of 5-aminosalicylates and FA in their formulations. However, there is only one published paper related to the simultaneous determination of MSZ and FA using the electroanalytical approach^19^. Up till now, no developed methods were reported for the simultaneous determination of all four 5-aminosalicylates and FA in a single analytical method.

The aim of this work is to apply different chromatographic techniques and biomimetic HPLC measurements to provide an insight into pharmacokinetic related drug properties. On the other hand, the development and validation of a single assay method for simultaneous determination of 5-aminosalicylates and FA in four in-house prepared FDCs will be approached according to the current guidelines defined by the pharmaceutical regulatory authorities. In the end, multiple 5-aminosalicylate and FA related drug products that are marketed in Europe for treatment of IBD will be tested using the developed method.

## Methods

### Reagents and chemicals

MSZ (100.0%), FA (90.7%) and OSZ (100.0%) sodium European Pharmacopoeia certified reference standards, BSZ disodium US Pharmacopoeia reference standard (100.0%), SASP British Pharmacopoeia Chemical Reference Substance (99.6%) and 5-acetylmesalazine internal standard (≥ 98.0%) were all provided by Sigma-Aldrich (St. Louis, MO, USA). Acetonitrile, ethanol, methanol and 2-propanol (all HPLC grade solvents), sodium hydroxide pellets (≥ 98.0%), formic acid for HPLC (98 – 100%), phosphate-buffered saline tablets (0.01 M phosphate buffer, 0.0027 M potassium chloride and 0.137 M sodium chloride, pH 7.4 at 25 °C), potassium phosphate monobasic and potassium phosphate dibasic (HPLC grade) were also obtained by Sigma-Aldrich. For preparation of *in-house* FDCs and quality control following products were purchased: Pentasa by Ferring Pharmaceuticals (Saint-Prex, Switzerland), Salofalk by Dr. Falk Pharma GmbH (Freiburg, Germany), Mesalazin-Kohlpharma by Kohlpharma GmbH (Merzig, Germany), Folacin by JGL (Rijeka, Croatia), Premid by Almirall S.A. (Barcelona, Spain), Salazopyrin by Recipharm Uppsala AB (Uppsala, Sweden) and Dipentum by Waymade PLC (Basildon, Essex, UK). Excipients for preparation of placebo used in method validation as well as its composition is presented in Supplementary Table S1. Placebo blend was prepared according to the information available in literature regarding the use of excipients in preparation of pharmaceutical products^20^.

### Stock and working solutions

Standard solutions of 5-aminosalicylates and FA for drug hydrophobicity and phospholipid affinity determination, frontal analysis and zonal elution studies were prepared in a concentration of 50 µM by dissolving the standards in 20 mM phosphate buffer (pH 7), as well as series of mobile phases containing FA in concentrations of 1, 5, 10, 15 and 20 µM. For the development of assay method, stock solutions of MSZ (250 µg/mL), FA (10 µg/mL) and internal standard (IS) (250 µg/mL) were prepared by dissolving the standards in ultrapure water (40 °C). Stock solutions of SASP (250 µg/mL), OSZ (125 µg/mL) and BSZ (375 µg/mL) were prepared by dissolving the standards in 5 mM NaOH. For method validation prepared placebo blend was dissolved in both water and 5 mM NaOH in the concentration of 1.0 mg/mL. Working solutions (mixtures of each 5-aminosalicylate with FA in the proposed ratio) were prepared by spiking the placebo solution with the standard solutions. The final working solutions contained 100 µg/mL of MSZ, 100 µg/mL of SASP, 50 µg/mL of OSZ, 150 µg/mL of BSZ, 100 µg/mL of IS and 0.2 µg/mL of FA. All solutions were freshly prepared before analysis, filtered through 0.20 μm polyethersulfone filters and stored in amber glassware.

### Preparation of in-house FDCs and samples for quality control of commercially available drugs

In-house FDCs used for method development were prepared by using commercially available 5-ASA drugs for treatment of IBD. Ten randomly chosen MSZ and SASP tablets and OSZ and BSZ capsules content were weighed and separately ground to a fine powder. For preparation of FDCs, an amount of powdered tablet or capsule equal to 500 mg of MSZ, 500 mg of SASP, 250 mg of OSZ and 750 mg of BSZ was mixed with the 1 mg of FA standard to achieve the desired ratio (Supplementary Table S2). The amount of each prepared FDC powder was weighed and transferred into an individual 10 mL amber volumetric flask and dissolved in water for MSZ/FA mixture and in 5 mM NaOH for other mixtures. An appropriate amount of IS solution was added, flasks were filled to the mark and sonicated for 15 min to provide complete solubilization. Solutions were centrifuged, filtered through a 0.2 µm polyethersulfone injection filter and diluted to achieve the concentrations equal to those of working solution described in previous section.

For quality control of commercially available 5-ASA and FA drugs, an amount of powdered tablets or capsules were weighed and transferred in 10 mL volumetric flask with the addition of IS and properly diluted with water for drugs containing MSZ and in 5 mM NaOH for other drugs to achieve the final concentrations of 100 µg/mL of MSZ, 100 µg/mL of SASP, 50 µg/mL of OSZ, 150 µg/mL of BSZ, 100 µg/mL of IS and 0.2 µg/mL of FA, which represents the 100% concentration level of calibration curve.

### RP-HPLC hydrophobicity method

All HPLC measurements were carried out on an Agilent 1100 series HPLC system (Agilent Technologies, Waldbronn, Germany) coupled with a diode array detector (DAD). System control, data collection and data processing were accomplished using ChemStation for LC 3D software by Agilent Technologies. RP-HPLC hydrophobicity determination was carried on Symmetry C18 reversed-phase column (150 × 4.6 mm, 3.5 µm particle size) obtained by Waters (Milford, MA, USA), with phosphate-buffered saline and methanol as mobile phase. The flow rate was set at 1.0 mL/min with the injection volume of 10.0 µL whilst the column temperature was maintained at 25.0 ± 0.1 °C. To obtain hydrophobicity, the log *k* values were calculated using retention times of investigated compounds (*t*_R_) and retention time of sodium nitrate as unretained compound (*t*_0_). All compounds were eluted starting with higher fractions of methanol in the mobile phase (60%) followed by measurements with 5% methanol reduction after each following run. At least five different concentrations of methanol in the mobile phase were applied for each standard substance and all measurements at each methanol fraction were performed in triplicates. For highly lipophilic compounds such as SASP, OSZ and BSZ measurement were performed until 25% of methanol in mobile phase due to the long retention times at lower methanol fractions in mobile phase, whilst measurements including MSZ and FA were carried until 5% and 10%, respectively. Column equilibration time of 20 min was necessary after every change of mobile phase. Finally, log *k*_w C18_ value, describing hydrophobicity, was obtained by calculating the *y*-intercept of the regression line in a plot of log *k* vs. methanol fraction in the mobile phase.

### Biomimetic studies

The affinity of analytes for immobilized phospholipids was measured using the immobilized phosphatidylcholine column (IAM.PC.DD2, 100 × 4.6 mm, 300 Å pore size, 10 μm particle size) which was obtained from Regis Technologies (Morton Grove, IL, USA). The same HPLC conditions, mobile phases and calculation approaches were used for evaluation of IAM affinity, as in determining hydrophobicity with the RP-HPLC column.

Binding percentage of analytes to plasma proteins (PPB_HSA_ and PPB_AGP_) was determined using the procedure described in our previously published paper^21^.

Frontal and zonal elution studies were carried out on HSA (Chiralpak-HSA, 50 × 4.6 mm, 5 μm particle size) biomimetic column obtained by ChromTech (Cedex, France). Elution of 5-aminosalicylates was achieved with a series of mobile phases consisting of 20 mM potassium phosphate buffer (pH 7.0) (A) and iso-propanol (B) with the FA addition of 0, 1, 5, 10, 15 and 20 µM. Isocratic elution with the flow rate of 1.8 mL/min for HSA was applied for all compounds, where for the elution of MSZ organic modifier was excluded due to the low retention of MSZ, whilst 30% (*v*/*v*) of iso-propanol in mobile phase was mandatory due to the unacceptably high retention times of 5-aminosalicylate prodrugs. Injection volume was set at 10.0 µL and the measurements were carried out at 25.0 ± 0.1 °C.

### Simultaneous determination of 5-aminosalicylates and FA

Same chromatographic system and software for data processing was used as in previous sections. Simultaneous chromatographic separation of 5-aminosalicylates and FA was achieved on an XBridge Phenyl column (150 × 4.6 mm, particle size 3.5 µm) obtained by Waters (Milford, MA, USA) maintained at 25.0 ± 0.1 °C with the flow rate of 1.0 mL/min. The mobile phase consisted of ultrapure water as mobile phase A and methanol as mobile phase B, both acidified with formic acid up to 0.2% (*v/v*). A sample volume of 10.0 µL was injected into the system and eluted using the following gradient program: 0***–***2 min, isocratic 5% B; 2***–***3 min, linear gradient 5***–***25% B; 3***–***7 min, isocratic 25% B; 7***–***9 min, linear gradient 25***–***70% B; 9***–***11 min linear gradient 70% B; 11***–***12 min, linear gradient 70***–***95% B; 12***–***16 min isocratic 95% B. Analysis time was 16 min while total run time was 20 min to allow re-equilibration of the stationary phase. MSZ and FA were detected at 300 and 285 nm respectively, whilst OSZ, SASP and BSZ were detected at 320 nm.

Method was validated according to ICH Q2 (R1) “Validation of analytical procedures: text and methodology”^22^ and Food and Drug Administration (FDA) “Analytical Procedures and Methods Validation for Drugs and Biologics”^23^. Solutions of placebo spiked with the standard solutions were used to replicate the matrix of the real sample. Visual examination of obtained chromatograms was performed and the peak purity factors were calculated. The linearity of the method was examined in the range of 50% up to 150% of nominal concentration on five concentration levels (50%, 75%, 100%, 125% and 150%). IS was used for calculating the surface ratio between analysed compound and IS and same principle was used through all calculations concerning peak area. Limit of detection (LOD) and limit of quantification (LOQ) were calculated as a signal to noise ratio 3:1 and 10:1, respectively. The precision of the method was studied as repeatability by analysing six individually prepared samples within the one day and intra-day precision by individually preparing and analyzing three samples each day for three consecutive days. All samples were analysed at 100% concentration level and results are expressed as RSD (%). The accuracy of the method was tested as a recovered amount of the analyte compared to the known concentration in the sample. Recoveries were tested on three levels; the lowest point is equal to 50%, medium to 100% and highest to 150% concentration level. Finally, small changes in temperature (± 1 °C), flow rate (± 5%), mobile phase composition (± 1%) and formic acid addition to mobile phase (± 0.05%) were applied to the method. Changes in retention times and peak areas were calculated to determine the robustness of the developed method and all changes were expressed as RSD (%).

All calculations regarding the method validation such as linearity, recovery and all RSD values were done using Microsoft Excel software. All data generated and analysed during this study are included in the article and in supplementary information file.

## Results and discussion

### RP-HPLC hydrophobicity and phospholipid-binding determination

All analytes, except MSZ, have shown strong interaction for the RP-C18 column. Linear relationships (*r* ≥ 0.99) between log *k* values and the volume fractions of methanol were found for all compounds. All log *k* values are the average of three measurements, with RSD (%) values lower than 0.99% (Table 1). Positive hydrophobicity parameters were obtained for all analytes with MSZ being highly hydrophilic (log *k*_w C18_ = 0.37) compared to all three prodrugs which have high hydrophobicity (log *k*_w C18_ ≥ 3.01), whilst FA has shown moderate hydrophobicity (log *k*_w C18_ = 0.99).

**Table 1:** Experimental parameters obtained by chromatographic techniques.

Analyte	log *k*_w C18_^a^	log *k*_w IAM_^a^	PPB_HSA_ (%)^b^	PPB_AGP_ (%)^b^
MSZ	0.37 ± 0.19	0.17 ± 0.02	61.44 ± 0.51	6.22 ± 1.08
FA	0.99 ± 0.31	− 0.37 ± 0.14	69.40 ± 0.53	3.45 ± 0.61
BSZ	3.01 ± 0.09	1.20 ± 0.11	91.64 ± 0.04	77.60 ± 0.58
SASP	3.71 ± 0.24	2.77 ± 0.23	93.62 ± 0.05	96.36 ± 0.13
OSZ	3.25 ± 0.09	1.69 ± 0.17	96.00 ± 0.03	90.33 ± 0.20

^a^log *k*_w C18_ and log *k*_w IAM_ values with the accompanied 95% confidence interval. ^b^PPB_HSA_ (%) and PPB_AGP_ (%) values with accompanied RSD (%).

On the other hand, the development of IAM chromatography added new perspectives for the use of chromatographic techniques in the profiling of new drugs, combining simulation of the cell membrane environment with rapid and reliable measurements. Our previous investigations encouraged us to apply this chromatographic system on 5-aminosalicylates and FA. Linear relationships (*r* ≥ 0.94) between log *k*_IAM_ values and the volume fraction of methanol were found for all compounds. The presented finding showed lower degree of interaction with phosphatidylcholine than with octadecyl group for both 5-aminosalicylates (log *k*_w IAM_ ≤ 2.77) and FA (log *k*_w IAM_ = -0.37). This phenomenon might be due to the presence of sterically exposed charged moieties on the phospholipid chains. Furthermore, a moderate linear correlation between hydrophobicity (log *k*_w C18_) and lipophilicity data (log *k*_w IAM_), obtained by HPLC, was observed (*r* = 0.91). It is possible that the unexpectedly high affinity of MSZ for the IAM column (log *k*_w IAM_ = 0.17) was due to its small size and consequently ability to penetrate within the phospholipid monolayer. All log *k* values are the average of three measurements with RSD (%) values lower than 3.55% (Table 1).

### Plasma protein binding studies

PPB has a noteworthy role in modulating the effective drug concentration at the pharmacological target. The mentioned concentration as well as consequential pharmacological activity of drugs may be influenced by co-administered drugs due to potential competition for the same binding site on plasma proteins.

HSA, the most abundant plasma protein, has binding sites capable of binding xenobiotics with a preference for acidic and neutral compounds. All obtained values are the average of three measurements (RSD values lower than 0.53%). High HSA binding affinity for all compounds (more than 61%) due to the presence of fully ionized carboxyl groups in the structure of investigated compounds (p*K*_a_ values of all compounds are not higher than 3.37) is very important finding (Table 1). On the other hand, AGP shows the preference for basic and neutral compounds. All obtained values are the average of three measurements (RSD values lower than 1.08%). According to obtained results, a high affinity for AGP, similar to that of HSA, was observed for SASP and OSZ (higher than 90%). Somewhat lower affinity for AGP than for HSA was found for BSZ (14% lower), while MSZ and FA show quite low affinity for AGP (Table 1). Furthermore, it is worth pointing out that the linear correlation in a plot of PPB_HSA_ vs. log *k*_w C18_ (*r* = 0.99), as well as in the plot of PPB_AGP_ vs. log *k*_w C18_ and (*r* = 0.98) was observed for 5-aminosalicylates and FA. The obtained results imply that hydrophobic forces are generally predominant in the binding mechanism. Drug binding to the HSA usually occurs in Sudlow site I or II, which is a hydrophobic cavity that is capable of holding multiple drug molecules. As well as HSA, AGP also has a wide central hydrophobic pocket that occurs as a main binding site for the ligand molecules^24,25^.

Observed high affinity of 5-aminosalicylates and FA for HSA protein led to further studies using frontal analysis and zonal elution methods to get an insight into FA binding on HSA and possible binding competition between FA and 5-aminosalicylates for the same place on HSA protein.

**Figure 2:**
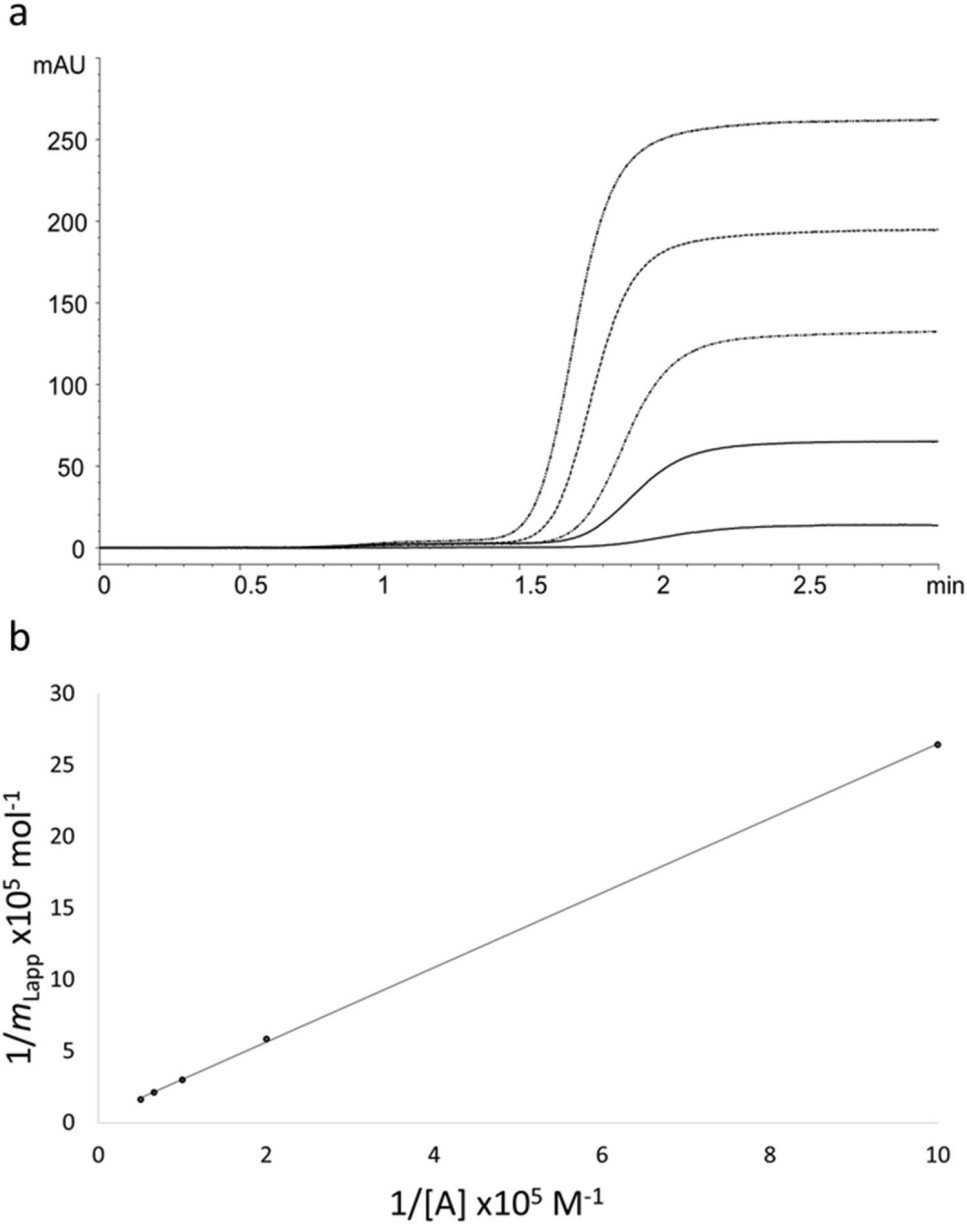
(**a**) Breakout curves obtained with frontal analysis, (**b**) double-reciprocal plot of apparent FA moles (1/*m*_*Lapp*_), versus applied concentration of FA (1/[A]).

Based on considerations above, frontal analysis was approached using FA which was run through the HSA column to monitor the saturation of FA specific binding sites. As the mobile phase containing FA is applied through the column, the detector signal increases due to the saturation of HSA binding sites, which results in a specific breakthrough curve. Breakthrough curves were obtained for each applied concentration (1, 5, 10, 15 and 20 µM) and were used to create a double-reciprocal plot of apparent FA moles required to reach the equilibrium (1/*m*_*Lapp*_*)*, vs. concentration of FA that was applied (1/[A]) as illustrated in Fig. 2a. Double-reciprocal plot (Fig. 2b) shows great linearity (r > 0.9999), even with high applied concentrations, implying that the FA has a single type of binding site on the HSA.

The slope and intercept of the obtained curve were used for calculation of the equilibrium constant (*K*_a_) and moles of binding sites (*m*_L_) for FA-HSA specific interaction, using the Eq. (1):

1 $$\frac{1}{{m}_{\mathrm{L}app}}=\frac{1}{{m}_{\mathrm{L}}\times {K}_{a}\times [\mathrm{A}]}+\frac{1}{{m}_{\mathrm{L}}}$$

The obtained value of moles of binding sites (*m*_L_ = 2.34 (± 1.16) × 10^–7^ mol) was further used for calculation of binding constant (*K*_a_) which equals 1.64 (± 0.03) × 10^4^ M^−1^; the values in brackets represent 95% confidence intervals. Hence, the obtained binding constant is specific for the conditions used and is determined using the HPAC method for the FA-HSA system for the first time. Previous studies using different techniques have shown comparable binding constants ranging from 0.53 to 9.7 × 10^4^ M^−1^^26–30^.

Having determined that FA has site-specific binding on HSA protein, a zonal competition study was conducted to examine the possible competitive binding between 5-aminosalicylates and FA. A mobile phase with FA in different concentrations (ranging from 0 to 20 µM, with 5 µM increments in each mobile phase) was run through the biomimetic column to saturate the FA specific binding sites. Upon saturation of all FA specific binding sites, a small fraction of examined compound, in this case each 5-aminosalicylate, was injected into the system at each applied FA concentration and the retention times were monitored. Obtained retention times were used to calculate the retention factor *k*, whose reciprocal value was used in a plot of 1/*k* vs. FA concentration in the mobile phase. Change in retention time with the increments of FA would imply that there is a competition for the same binding site on HSA between FA and 5-aminosalicylates. Linear dependency (r > 0.991) was obtained with a positive slope for each 5-aminosalicylate. Linear correlation implies that there is no positive or negative allosteric modulation on HSA protein due to the binding of FA. Furthermore, low values of the slopes (0.0004–0.0019) imply that the competition of the 5-aminosalicylates for the same binding site is very low. With the statistical analysis of the slope of the regression line, whether it is equal or different from zero we can confirm the above stated. With the regression analysis (95% confidence interval) all obtained *P*-values of slopes were higher than 0.05, ranging from 0.09 to 0.13. With *P*-values > 0.05 we can accept the null hypothesis, stating that the slope is not different from 0. This could be explained by the fact that FA binds to a different site on HSA compared to the rest of 5-aminosalicylates. According to the findings in the literature, FA binds to a site located in Domain I on HSA protein whilst SSZ binds to Domain IIb and BSZ and MSZ bind to Domain IIIa^31–33^. To the best of our knowledge, this is the first study on possible competitive binding between 5-aminosalicylates and FA using frontal analysis approach. Altogether these results indicate that the obtained results are in favour of further development of fixed-dose combination implying that there will be no interactions of FA with any of 5-aminosalicylates if taken simultaneously in a single formulation.

### Simultaneous assay method development

Our preliminary study has shown that column chemistry and mobile phase composition will have a major impact on method performance, such as peak resolution, retention time and tailing factor, due to the considerable difference in analyte hydrophobicity. Reversed-phase columns, XBridge C18, 150 × 4.6 mm, particle size 5 µm column by Waters and Zorbax SB C8, 150 × 4.6 mm, particle size 5 µm by Agilent Technologies, were tested. Due to the high polarity of MSZ, poor retention was observed (*k* = 0.08) with gradient elution starting with low levels of organic solvent (less than 5% of methanol) in the mobile phase, whilst high hydrophobicity of prodrugs resulted in a wide peak with prominent peak tailing (tailing factor, TF > 3.87). Best results in terms of retention factor, reproducibility, complete separation and peak shape were obtained with XBridge Phenyl (150 × 4.6 mm, particle size 5 µm) by Waters. The π–π interaction between phenyl groups bonded on silica and aromatic rings present in the analytes resulted in higher retention of MSZ. Therefore, this column was chosen as the most suitable for future research.

**Figure 3:**
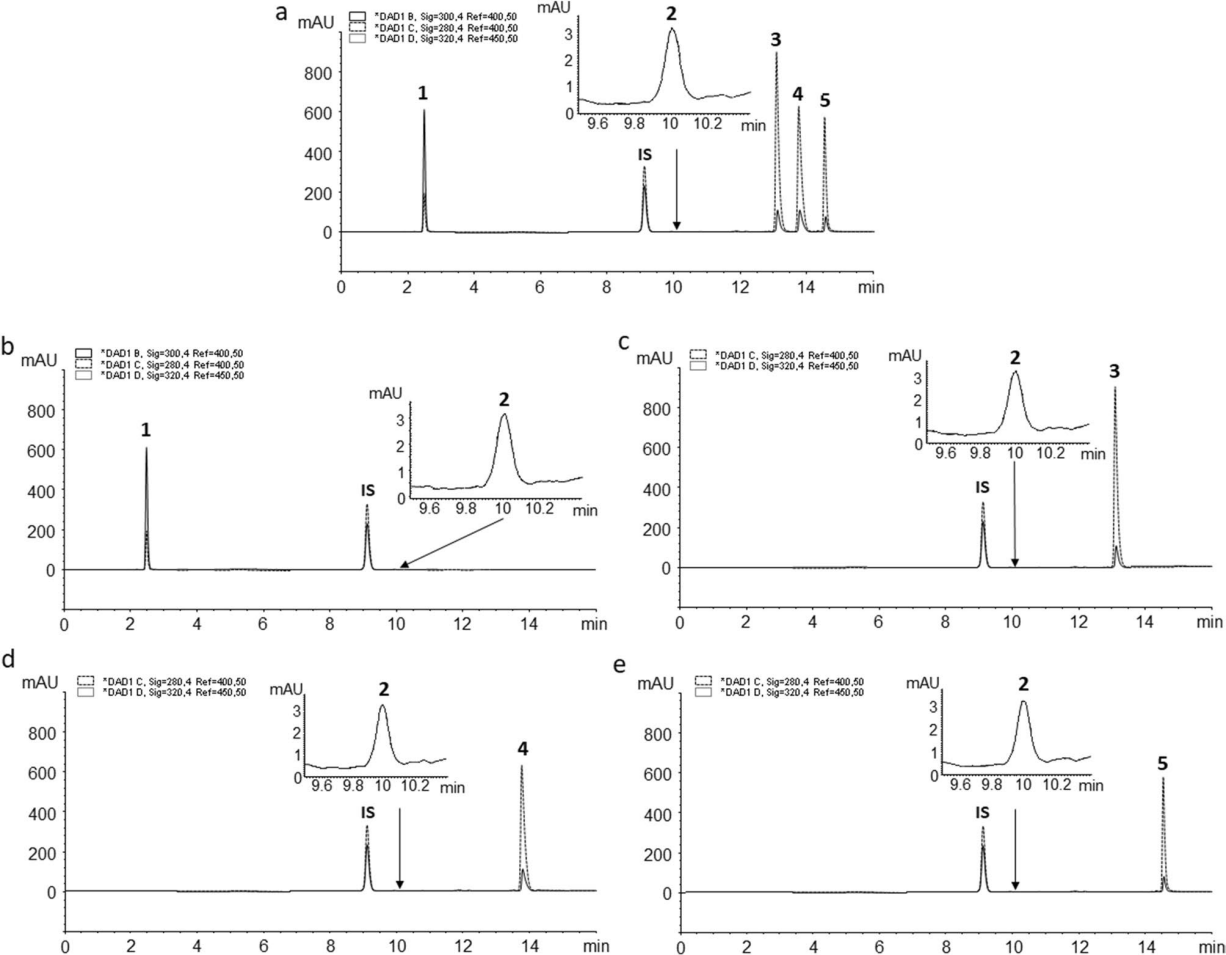
Chromatograms of: (**a**) mixed standard solution including MSZ (1), FA (2), BSZ (3), SASP (4), OSZ (5) and IS; and in-house FDCs (**b**) MSZ + FA; (**c**) BSZ + FA; (**d**) SASP + FA and (**e**) OSZ + FA at 100% concentration level (Chromatographic conditions: XBridge Phenyl column (150 × 4.6 mm, particle size 3.5 µm) and the mobile phase consisted of ultrapure water (A) and methanol (B), both acidified with formic acid up to 0.2% (*v/v*)).

Gradient elution, starting with a low percentage of organic modifier (5%), was necessary to achieve satisfactory retention of MSZ (*k* = 0.248) but also to elute prodrugs in acceptable time (*k* ≥ 6.078). As it was expected, acidified mobile phase (0.2% (*v*/*v*) formic acid, pH = 2.34), compared to neutral or alkali, had the advantage of creating an environment in which retention of polar acidic analyte such as MSZ (*k* = 0.337) was more easily achieved. Peak tailing of all peaks, including prodrugs, was minimized (TF < 2.26) as well as resolution maximized (*R*s > 3.67). Therefore, a 0.2% (*v*/*v*) addition of formic acid was chosen as an additive in both mobile phases. Optimal wavelengths were chosen for the detection of each analyte: MSZ was detected at 300 nm, IS, BSZ, SASP and OSZ at 320 nm and FA at 285 nm. Injection volume of 10.0 µL was chosen as optimal to achieve the best sensitivity without overloading the column or detector. To minimise the error of the injector IS was introduced. The chromatograms of mix standard solutions and each 5-aminosalicylate/FA FDC are presented in Fig. 3. System suitability test, as proof of method quality and applicability, was used by analysing seven replicate injections of standard solutions at 100% concentration level to determine the parameters such as retention and relative retention time, number of theoretical plates, retention factor and tailing factor (Supplementary Table S3).

### Method validation

As mentioned above the method was validated according to the current ICH Q2(R1) and FDA guidelines^22,23^. Solutions of placebo spiked with the standard solutions were used for method validation. Satisfactory resolution between adjacent peaks was achieved as resolution factor was higher than 1.5 All peak purity factor values were greater than 999.7 and it was observed that there are no interferences between excipients in placebo solution and analyte peaks. Correlation coefficients obtained by linear regression analysis were all above 0.9997 indicating satisfactory linearity of method. Low LOD (0.01 µg/mL) and LOQ (0.03 µg/mL) values indicate that method is suitable for quantifying low concentrations of FA (Table 2).

**Table 2:** Method calibration data.

Analyte	Linearity range (µg/mL)	Equation	*r*^a^	*s*_E_^b^	LOD (µg/mL)^c^	LOQ (µg/mL)^d^
MSZ	50–150	*y* = 0.0120 *x* + 0.0079	0.9999	0.0063	0.10	0.33
FA	0.1–0.3	*y* = 0.0533 *x* + 0.0012	0.9997	0.0001	0.01	0.03
BSZ	75–225	*y* = 0.0196 *x* + 0.0024	0.9997	0.0246	0.05	0.16
SASP	50–150	*y* = 0.0261 *x* + 0.0210	0.9998	0.0181	0.04	0.14
OSZ	25–75	*y* = 0.0261 *x* + 0.0130	0.9999	0.0037	0.02	0.08

^a^*r*—Pearson correlation coefficient. ^b^*s*_E_—standard error of estimate. ^c^LOD calculated as a signal to noise ratio (3:1). ^d^LOQ calculated as a signal to noise ratio (10:1).

All samples for precision evaluation were prepared as described in section “Stock and working solutions” and analysed at 100% concentration level (results are expressed as RSD (%) (Supplementary Table S4)). Obtained RSD (%) values were lower than 0.70 for 5-aminosalicylates and lower than 2.29 for FA, implying that the method satisfies the repeatability test but also remains precise during the longer period. Slightly higher RSD (%) values related to FA are due to its low concentration in prepared samples. Accuracy of the method was expressed by calculating the recovery percentage on three levels as described above. Consistent analytical recovery (98.9–101.8%; RSD ≤ 2.6%) was obtained over the investigated concentration range implying that the method is accurate in the whole range of the calibration curve (Supplementary Table S4).

The analytical method robustness was expressed as RSD (%) values of changes in retention times and peak areas. Put together, all obtained values of RSD (%) were below 4.87% implying that selected analytical parameters did not affect precision and accuracy of the method.

### Analysis of marketed drugs

Finally, the developed method was successfully applied to determine the assay of MSZ, BSZ, SASP, OSZ and FA in tablet and capsule formulations available in Europe that are used in the treatment of IBD. Recoveries were expressed as a percentage of the recovered sample compared to the labelled claim. All obtained results are presented in Table 3. Thus, the results of analysis of market drugs (recoveries range from 98.7 to 102.5% with the RSD (%) ≤ 1.9) evidenced high extraction efficiency, reproducibility and reliability of the novel method. It is evident from these results that assay of investigated formulations of MSZ, SASP and FA lie within the limits specified in British Pharmacopeia (BP) requirements (95.0–105.0% of the stated amount)^34^. It is important to emphasize that assay results obtained from the analysis of BSZ capsules were well within the limits specified in United States Pharmacopoeia (USP) (90.0–110.0%)^35^. On the other side, OSZ does not have a finished product monography in either BP or USP.

**Table 3:** Results of analyses of marketed formulations (*n* = 3).

Commercial formulation	Manufacturer	Active substance	Labelled amount (mg)	Found amount (mg)	Found/labelled (%)	RSD (%)
Pentasa	Ferring Pharmaceuticals, Saint-Prex, Switzerland	MSZ	500	499.50	99.9	1.6
Salofalk	Dr. Falk Pharma GmbH, Freiburg, Germany	MSZ	500	493.70	98.7	1.5
Mesalazin-Kohlpharma	Kohlpharma GmbH, Merzig, Germany	MSZ	500	511.60	102.3	0.2
Folacin	JGL, Rijeka, Croatia	FA	5	4.99	99.7	1.6
Premid	Almirall S.A., Barcelona, Spain	BSZ	750	756.75	100.9	1.9
Salazopyrin	Recipharm Uppsala AB, Uppsala, Sweden	SASP	500	510.55	102.1	0.6
Dipentum	Waymade PLC, Basildon, Essex, UK	OSZ	500	512.30	102.5	1.9

The developed and applied method provides a simple solution for analysis of multiple 5-aminosalicylates and folic acid within a single run, thus eliminating the exceptionally time-consuming process of mobile phase change and system preparation as it would be necessary if different methods for each analyte were used, which is also in accordance with the principles of green chemistry. The simplicity of the method lays in the usage of non-buffered mobile phases that are easily prepared, which makes this method usable with mass spectrometry, as well as in its capability of analysing multiple samples with complex matrices consisting of various excipients without compromising method selectivity.

## Conclusions

With the use of conventional and biomimetic columns an insight into pharmacokinetic related drug properties was achieved. High correlations between measured hydrophobicity values and drug permeability and PPB were obtained. The obtained drug properties provided useful information for upcoming development stages such as formulation optimization and in vitro pharmacokinetic studies. FA was successfully characterized regarding the HSA binding properties using frontal analysis and no interactions between FA and any of the 5-aminosalicylates were observed with the zonal elution studies. Furthermore, the analytical method for assay determination in proposed FDCs was successfully developed, including easy and simple sample and mobile phase preparation. The developed method was successfully validated and applied to in-house prepared FDCs as well as to commercially available products marketed in the European Union for IBD treatment. With further studies, proposed 5-aminosalycilates and FA based FDCs could find potential application in treatment of inflammatory bowel diseases.

## Supplementary information

Supplementary Information.

## Data Availability

All data generated or analysed during this study are included in this published article [and its supplementary information files].

## References

[1] Hnatyszyn A (2019). Colorectal carcinoma in the course of inflammatory bowel diseases. Hered. Cancer Clin. Pract..

[2] Hvas CL, Bendix M, Dige A, Dahlerup JF, Agnholt J (2018). Current, experimental, and future treatments in inflammatory bowel disease: a clinical review. Immunopharmacol. Immunotoxicol..

[3] Burr N, Hull M, Subramanian V (2017). Folic acid supplementation may reduce colorectal cancer risk in patients with inflammatory bowel disease. J. Clin. Gastroenterol..

[4] Gomollón F, Gisbert JP (2009). Anemia and inflammatory bowel diseases. World J. Gastroenterol..

[5] World Health Organization. Guidelines for registration of fixed-dose combination medicinal products. *WHO Technical Report Series* No. 929 (2005).

[6] Hens B (2019). “Development of fixed dose combination products” workshop report: considerations of gastrointestinal physiology and overall development strategy. AAPS J..

[7] Bunally S, Young RJ (2018). The role and impact of high throughput biomimetic measurements in drug discovery. ADMET DMPK.

[8] Joseph KS, Hage DS (2010). Characterization of the binding of sulfonylurea drugs to HSA by high-performance affinity chromatography. J. Chromatogr. B Anal. Technol. Biomed. Life Sci..

[9] Anguizola J, Bi C, Koke M, Jackson A, Hage DS (2016). On-column entrapment of alpha1-acid glycoprotein for studies of drug-protein binding by high-performance affinity chromatography. Anal. Bioanal. Chem..

[10] Hage DS (2002). High-performance affinity chromatography: a powerful tool for studying serum protein binding. J. Chromatogr. B Anal. Technol. Biomed. Life Sci..

[11] Patel KM, Patel CN, Panigrahi B, Parikh AS, Patel HN (2010). Development and validation of spectrophotometric methods for the estimation of mesalamine in tablet dosage forms. J. Young Pharm..

[12] Lebiedzińska A, Da̧browska M, Szefer P, Marszałł M (2008). High-performance liquid chromatography method for the determination of folic acid in fortified food products. Toxicol. Mech. Methods.

[13] Rafael JA (2007). Validation of HPLC, DPPH• and nitrosation methods for mesalamine determination in pharmaceutical dosage forms. Rev. Bras. Ciencias Farm. J. Pharm. Sci..

[14] Sahoo NK, Sahu M, Srinivasa Rao P, Ghosh G (2013). Validation of stability indicating RP-HPLC method for the estimation of mesalamine in bulk and tablet dosage form. Pharm. Methods.

[15] Elmasry MS (2011). Quantitative HPLC analysis of mebeverine, mesalazine, sulphasalazine and dispersible aspirin stored in a Venalink monitored dosage system with co-prescribed medicines. J. Pharm. Biomed. Anal..

[16] Nigović B, Šimunić B (2003). Determination of 5-aminosalicylic acid in pharmaceutical formulation by differential pulse voltammetry. J. Pharm. Biomed. Anal..

[17] Ramezani Z, Dibaee N (2012). Determination of sulfasalazine in sulfasalazine tablets using silver nanoparticles. Iran. J. Pharm. Sci..

[18] Patelia EM (2013). Estimation of Balsalazide By HPTLC-densitometry method in pharmaceutical formulation. J. Chromatogr. Sep. Tech..

[19] Nigović B, Mornar A, Brusač E, Jeličić M-L (2019). Selective sensor for simultaneous determination of mesalazine and folic acid using chitosan coated carbon nanotubes functionalized with amino groups. J. Electroanal. Chem..

[20] Rowe RC, Sheskey PJ, Quinn ME (2009). Handbook of Pharmaceutical Excipients.

[21] Brusač E (2019). Pharmacokinetic profiling and simultaneous determination of thiopurine immunosuppressants and folic acid by chromatographic methods. Molecules.

[22] ICH. International Conference on Harmonisation (ICH) of Technical Requirements for the Registration of Pharmaceuticals for Human Use (2005) Validation of Analytical Procedures: Text and Methodology Q2 (R1), pp 1–13 (2005).

[23] Food and Drug Administration (FDA)—Analytical Procedures and Methods Validation for Drugs and Biologics Guidance for Industry Analytical Procedures and Methods Validation for Drugs and Biologics, Guidance for Industry (2015), pp 1–18.

[24] Zsila F, Iwao Y (2007). The drug binding site of human α1-acid glycoprotein: insight from induced circular dichroism and electronic absorption spectra. Biochim. Biophys. Acta Gen. Subj..

[25] Yang F, Zhang Y, Liang H (2014). Interactive association of drugs binding to human serum albumin. Int. J. Mol. Sci..

[26] Śliwińska-Hill U, Wiglusz K (2019). Multispectroscopic studies of the interaction of folic acid with glycated human serum albumin. J. Biomol. Struct. Dyn..

[27] Chilom CG, Bacalum M, Stanescu MM, Florescu M (2018). Insight into the interaction of human serum albumin with folic acid: a biophysical study. Spectrochim. Acta Part A Mol. Biomol. Spectrosc..

[28] Jha NS, Kishore N (2011). Thermodynamic studies on the interaction of folic acid with bovine serum albumin. J. Chem. Thermodyn..

[29] Bourassa P, Hasni I, Tajmir-Riahi HA (2011). Folic acid complexes with human and bovine serum albumins. Food Chem..

[30] Panja S, Khatua DK, Halder M (2018). Simultaneous binding of folic acid and methotrexate to human serum albumin: insights into the structural changes of protein and the location and competitive displacement of drugs. ACS Omega.

[31] Bourassa P, Chanphai P, Tajmir-Riahi HA (2017). Folic acid delivery by serum proteins: loading efficacy and protein morphology. J. Biomol. Struct. Dyn..

[32] Shahabadi N, Fili SM (2014). Molecular modeling and multispectroscopic studies of the interaction of mesalamine with bovine serum albumin. Spectrochim. Acta Part A Mol. Biomol. Spectrosc..

[33] Zsila F (2013). Subdomain IB is the third major drug binding region of human serum albumin: toward the three-sites model. Mol. Pharm..

[34] Medicines and Healthcare Products Regulatory Agency. British Pharmacopoeia 2020; Medicines and Healthcare Products Regulatory Agency: London, UK, 2020. (used to access the monographs of the following finished products: Mesalazine Gastro-resistant Tablets, Folic Acid Tablets and Sulfasalazine Gastro-resistant Tablets).

[35] United States Pharmacopoeia 42—National Formulary 37; Published on Jun 03, 2019; United States Pharmacopoeial Convention: Rockville, MD, USA, 2018. (used to access the monographs for the following finished products: Mesalamine Delayed-Release Tablets (page 2762), Balsalazide Disodium Capsules (page 468), Folic Acid Tablets (page 1981), Sulfasalazine Delayed-Release Tablets (page 4129).

