# Supplementary material for: A chromatographic approach to development of 5-aminosalicylate/folic acid fixed-dose combinations for treatment of Crohn’s disease and ulcerative colitis
Source: Sci Rep. 2020 Nov 30;10:20838. doi: 10.1038/s41598-020-77654-2 (PMC7705649; doi:10.1038/s41598-020-77654-2)
Supplement: Supplementary file 1 — Supplementary Information. [file 41598_2020_77654_MOESM1_ESM.docx]

A chromatographic approach to development of 5-aminosalicylate/folic acid fixed-dose combinations for treatment of Crohn’s disease and ulcerative colitis

Mario-Livio Jeličić ^1^, Edvin Brusač ^1^, Daniela Amidžić Klarić ^1^, Biljana Nigović ^1^, Nikša Turk ^2^ and Ana Mornar ^1*^

^1^ University of Zagreb, Faculty of Pharmacy and Biochemistry, A. Kovačića 1, 10000 Zagreb, Croatia

^2^ Clinical Hospital Center Zagreb, Kišpatićeva 12, 10000 Zagreb, Croatia

Corresponding author: Ana Mornar, PhD

E-mail: [amornar@pharma.hr](mailto:amornar@pharma.hr) ; Tel.: +385-1-481-8288

University of Zagreb, Faculty of Pharmacy and Biochemistry,

A. Kovačića 1, 10000 Zagreb, Croatia

**Table S1** Placebo composition

| Excipient | Mass fraction range ^a^  (% w/w) | Placebo composition  (% w/w) |
| --- | --- | --- |
| Calcium stearate | < 1.0 | 1.0 |
| Celulose (microcrystalline) | 5.0 – 15.0 | 10.0 |
| Croscarmellose sodium | 0.5 – 5.0 | 3.0 |
| Crospovidone | 2.0 – 5.0 | 3.5 |
| Ethylcellulose | 1.0 – 3.0 | 2.0 |
| Eudragit E | / | 5.0 |
| Eudragit L | / | 5.0 |
| Glycine | / | 5.0 |
| Hypromellose | 2.0 – 5.0 | 3.5 |
| Lactose hydrate | / | 7.0 |
| Macrogol | / | 5.0 |
| Magnesium stearate | 0.25 – 5.00 | 2.5 |
| Maize starch | 3.0 – 15.0 | 10.0 |
| Povidone | 0.5 – 5.0 | 3.0 |
| Silicon dioxide (colloidal) | 2.0 – 10.0 | 6.0 |
| Sodium carbonate (anhydrous) | / | 5.0 |
| Sodium CMC | 1.0 – 6.0 | 6.0 |
| Talc | 5.0 – 30.0 | 17.5 |

^a^ mass fraction range used in preparation of drug formulations as per Handbook of pharmaceutical excipients ^20^. Excipients for which mass fraction was not defined were added in the range from 5.0 – 7.0 %.

**Table S2** Detailed description of prepared fixed dose combinations

| Market name | Drug product used ^a^ | API 1 | API 2 | Amount of API 1 (mg) | Amount of  API 2 (mg) | Excipients ^b^ |
| --- | --- | --- | --- | --- | --- | --- |
| FDC 1.1 | Pentasa | MSZ | FA | 500 | 1 | Cellulose (microcrystalline), Ethylcellulose, Magnesium stearate, Povidone & Talc |
| FDC 1.2 | Salofalk | MSZ | FA | 500 | 1 | Calcium stearate, Cellulose (microcrystalline), Croscarmellose sodium, Eudragit E, Eudragit L, Glycine, Hypromellose, Macrogol, Povidone, Silicon dioxide (colloidal), Sodium carbonate (anhydrous) & Talc |
| FDC 1.3 | Mesalazin-Kohlpharma | MSZ | FA | 500 | 1 | Eudragit E, Eudragit L, Cellulose (microcrystalline), Glycine, Povidone, Silicon dioxide (colloidal), Sodium carbonate (anhydrous) & Talk |
| FDC 2 | Premid | BSZ | FA | 750 | 1 | Magnesium stearate & Silicon dioxide (colloidal) |
| FDC 3 | Salazopyrin | SASP | FA | 500 | 1 | Macrogol, Magnesium stearate, Maize starch, Povidone & Silicon dioxide (colloidal) |
| FDC 4 | Dipentum | OSZ | FA | 250 | 1 | Magnesium stearate |

**^a^** powdered drug product used for preparation of fixed dose combination by mixing with FA standard

^b^ excipients present in the drug products as well as in prepared FDC

**Table S3** System suitability data

| **Analyte** | **Parameter (mean value ± standard deviation), n = 7** | | | | | | |
| --- | --- | --- | --- | --- | --- | --- | --- |
|  | ***t*_R_ (min) ^a^** | ***Rt*_R_ ^b^** | ***Rs* ^c^** | ***k* ^d^** | **N ^e^** | **TF ^f^** | **A ^g^** |
| MSZ | 2.47 ± 0.01 | 0.269 ± 0.001 | / | 0.337 ± 0.002 | 8671 ± 230 | 1.33 ± 0.01 | 2378 ± 3 |
| IS | 9.16 ± 0.01 | 1.000 | 5.69 ± 0.05 | 3.952 ± 0.004 | 36144 ± 765 | 1.33 ± 0.01 | 2004 ± 1 |
| FA | 10.02 ± 0.01 | 1.095 ± 0.001 | 4.93 ± 0.04 | 4.417 ± 0.005 | 68342 ± 870 | 1.06 ± 0.04 | 16.34 ± 0.14 |
| BSZ | 13.09 ± 0.01 | 1.431 ± 0.001 | 19.70 ± 0.30 | 6.078 ± 0.002 | 109024 ± 48 | 2.20 ± 0.04 | 5972 ± 9 |
| SASP | 13.75 ± 0.01 | 1.502 ± 0.001 | 3.67 ± 0.01 | 6.431 ± 0.004 | 77228 ± 722 | 2.36 ± 0.01 | 5018 ± 14 |
| OSZ | 14.53 ± 0.01 | 1.588 ± 0.002 | 4.88 ± 0.06 | 6.854 ± 0.001 | 232086 ± 5988 | 1.74 ± 0.02 | 2959 ± 20 |

^a^ *t*_R_ – retention time

^b^ *k* – relative retention time

^c^ *Rs* – resolution

^d^ *k* – capacity factor

^e^ N – theoretical plates

^f^ TF – USP tailing factor

^g^ A – peak area

Table S4 Intra- and inter-day assay precision and accuracy

| **Analyte** | **Precision as RSD (%)** | | **Accuracy as recovery ± RSD (%)** | | |
| --- | --- | --- | --- | --- | --- |
|  | **Intra-day precision (*n* = 6) ^a^** | **Inter-day**  **precision (*n* = 9) ^b^** | **low**  **(n = 3) ^c^** | **medium**  **(n = 3) ^d^** | **high**  **(n=3) ^e^** |
| MSZ | 0.20 | 0.27 | 101.8 ± 0.7 | 99.0 ± 0.2 | 101.2 ± 0.6 |
| FA | 1.41 | 2.29 | 100.0 ± 2.6 | 99.6 ± 2.0 | 100.6 ± 0.9 |
| BSZ | 0.20 | 0.70 | 100.7 ± 0.1 | 100.1 ± 0.3 | 99.8 ± 0.4 |
| SASP | 0.21 | 0.58 | 98.9 ± 0.3 | 100.6 ± 0.5 | 100.2 ± 0.3 |
| OSZ | 0.16 | 0.40 | 99.8 ± 0.8 | - 1. ± 0.3 | 99.2 ± 0.4 |

^a^ Intra-day precision was calculated by analyzing six individually prepared samples at 100% concentration level within the one day

^b^ Inter-day precision was calculated by individually preparing and analyzing three samples at 100% concentration level each day for three consecutive days

^c^ Accuracy was calculated by individually preparing and analyzing three samples at 50% concentration level

^d^ Accuracy was calculated by individually preparing and analyzing three samples at 100% concentration level

^e^ Accuracy was calculated by individually preparing and analyzing three samples at 150% concentration level
